# Protocol and reference values for minimal detectable change of MyotonPRO and ultrasound imaging measurements of muscle and subcutaneous tissue

**DOI:** 10.1038/s41598-022-17507-2

**Published:** 2022-08-11

**Authors:** Paul E. Muckelt, Martin B. Warner, Tom Cheliotis-James, Rachel Muckelt, Maria Hastermann, Britt Schoenrock, David Martin, Robert MacGregor, Dieter Blottner, Maria Stokes

**Affiliations:** 1 School of Health Sciences, University of Southampton, Southampton, UK; 2 Centre for Sport, Exercise and Osteoarthritis Research Versus Arthritis, Southampton, UK; 3 University Centre Sparsholt, Winchester, UK; 4 Corporate Member of Freie Universität Berlin, Humboldt-Universität zu Berlin, Experimental and Clinical Research Center (ECRC) & NeuroCure Clinical Research Center (NCRC), Clinical Neuroimmunology, Charité–Universitätsmedizin Berlin, Lindenbergerweg 80, 13125 Berlin, Germany; 5 NeuroMuscular Group, Center of Space Medicine and Extreme Environments at Charité, Berlin, Germany; 6 KBR, Houston, TX USA; 7 Airbus DS Space Systems, Inc, Houston, TX USA; 8 Southampton NIHR Biomedical Research Centre, Southampton, UK; 9 Charité–Universitätsmedizin Berlin, Corporate Member of Freie Universität Berlin, Humboldt-Universität zu Berlin, Berlin Institute of Health, Institute of Integrative Neuroanatomy, Berlin, Germany

**Keywords:** Physiology, Medical research, Medical imaging

## Abstract

The assessment of muscle health is of paramount importance, as the loss of muscle mass and strength can affect performance. Two non-invasive tools that have been found to be useful in this are the MyotonPRO and rehabilitative ultrasound imaging, both have shown to be reliable in previous studies many of which conducted by the research team. This study aims to determine the reliability of previously unassessed local body structures and to determine their minimal detectable changes (MDC) to support both researchers and clinicians. Twenty healthy participants were recruited to determine the reliability of seven skin positions out of a previously established protocol. Reliability was determined between three independent raters, and day to day reliability was assessed with one rater a week apart. Intraclass Correlation Coefficients (ICC) between raters and between days for tissue stiffness, tone and elasticity range from moderate to excellent (ICC 0.52–0.97), with most good or excellent. ICCs for subcutaneous thickness between days was good or excellent (ICC 0.86–0.91) and moderate to excellent between raters (ICC 0.72–0.96), in muscles it was moderate to excellent between raters and days (ICC 0.71–0.95). The protocol in this study is repeatable with overall good reliability, it also provides established MDC values for several measurement points.

## Introduction

The assessment of muscle health is of paramount importance, as the loss of muscle mass and strength can affect physical function^1^, quality of life^2^, and even mortality^3^. Assessment is relevant to a variety of situations and settings, many of which are out in the field where ease of access, handling and cost are critical factors. These situations include assessing changes in muscle over time in people: who are frail due to ageing^4^; after injury^5^; post-surgery^6^; during a period of inactivity (e.g. on intensive care^7^; neurodegenerative diseases i.e. Parkinson’s disease, stroke^8^ etc.; or during/after exposure to microgravity^9^). Accurate and reliable assessment requires robust protocols using valid and reliable tools. Assessment of abnormality for an individual can be made by comparison with normative values in the literature or between groups of people^10^. Where individuals are assessed over time, the precision of error in measurements needs to be known, so that real change can be identified, hence the minimal detectable change (MDC) for a measurement is needed^10–12^. The present paper describes a protocol for a study of astronauts (Myotones project; European Space Agency) on 6-month missions on the International Space Station (ISS), to examine whether there are any effects of microgravity on muscles/tendons/fascial biomechanical and viscoelastic tissue properties. The structures under investigation in the present reliability study were selected by anatomical criteria for their superficial location, ease of recognition of anatomical landmarks on the body for locating measurement points (MPs), and ease of reference for operators without a medical background. The study focuses on sites not previously evaluated for reliability and provides MDC values for each site, to enable individuals to be assessed and monitored over time.

Two portable technologies that have been shown to be valid and reliable and being used in the Myotones project are rehabilitative Ultrasound Imaging (RUSI) for measuring muscle thickness^13,14^ and non-invasive Myoton technology for assessing biomechanical and viscoelastic properties^15,16^. There is evidence of good reliability for both RUSI^17–19^ and the MyotonPRO device^8,11^ for particular muscles and tendons, but for new sites used in the Myotones project, their reliability (both interrater and intrarater) needs to be established.

The RUSI technique provides a safe, portable and relatively inexpensive means to evaluate muscle health status and observe changes in clinical and research environments^13,14^. Muscle strength is correlated to cross-sectional area (CSA)^20^ and CSA is strongly correlated with muscle thickness^21,22^. Muscle thickness therefore provides an indirect measure of muscle strength without the need for complex equipment and in people not otherwise able to perform strength tests, e.g., due to pain or cognitive impairments.

The MyotonPRO device does not require such high levels of expertise as RUSI and can be used effectively by novices^16^, as long as the correct measurement positions can be found. The MyotonPRO detects the damped natural oscillations of the superficial tissue in the form of an acceleration signal allowing resting tone (Hz), dynamic stiffness (N/m) and elasticity (logarithmic decrement; log), to be measured^16^.

The present study examined the reliability of the MyotonPRO and RUSI measurements at specific sites for various structures (muscle, tendon, fascia) of the lower and upper limbs and the trunk as previously reported in principle from a recent study on 24 healthy male bedrest participants^23^, to produce MDC values for monitoring changes over time in individuals. These sites are based on the structures being assessed in the Myotones project on the ISS, which aims to evaluate a means of rapid, efficient, accurate inflight monitoring of the health status of an astronaut’s muscles and related soft tissues^24^. Assessing these structures reliably is also intended to be of use to clinicians and researchers.

## Aim

To describe the protocol for examining several muscles potentially affected by prolonged inactivity affected by inactivity using two technologies (MyotonPRO and ultrasound imaging) and to establish reliability of measurements and produce MDC values.

## Objectives

Define the methodology and protocol used to measure the mechanical properties of superficial structures using the MyotonPRO and muscle and subcutaneous tissue thickness using ultrasound imaging.

To examine interrater reliability of Myoton and ultrasound measurements between three independent researchers at seven measurement sites on the same day.

To examine intrarater reliability of measurements taken on different days one week apart.

To establish MDC values for measurements of the various structures, repeated on different days to provide reference values for assessment of change in individuals over time.

## Methods

### Participants

Published recommendations of sample size requirements for reliability studies vary, but 15–20 participants has been suggested as sufficient^25^, and previous studies with Myoton have used 20 or fewer participants^26–28^. Twenty healthy participants were recruited (n = 10 males, n = 10 females) from staff and students at the University of Southampton (age 28.95 ± 2.77 BMI 24.28 ± 1.47) took part in the study. Ethics approval was obtained from the Faculty of Environmental and Life Sciences Ethics Committee (no. 40307), University of Southampton. All methods were performed in accordance with the relevant guidelines and regulations. Posters were circulated throughout the faculty advertising the study. Informed consent was obtained from all subjects prior to testing. Participants were asked to attend two sessions, the first lasting approximately two hours, and the second a week later lasting one hour held in the afternoons^29^. The room was set to an ambient temperature 22–24 °C, warm clothes worn during measurements, to avoid temp induced tonus changes.

#### Inclusion criteria

Over the age of 18 up to the age of 40; able to understand English.

#### Exclusion criteria

Musculoskeletal injury or surgery in the last five years which led to immobility for more than one week; uncontrolled diabetes or blood pressure; a known neurological disorder; arthritis restricting ability to perform everyday activities; receiving treatment for cancer; taking medication which affects muscle function. No scars on MP sites, no obvious inflammation/reddening of the skin, no underlying disease leading to spasms/paralysis (Parkinson’s disease, Huntington’s disease, stroke, nerve damage, etc.), no tonus reducing or enhancing agents were taken 12 h previous to the measurement (muscle relaxants, coffee more than 1 cup, alcohol, drugs, etc.), Participants were asked not to undertake any strenuous exercise within 12 h before the data collection session, including their mode of transport to the session (e.g. to take public transport or drive, as opposed to walking or cycling).

### Measurement points

**Figure 1:**
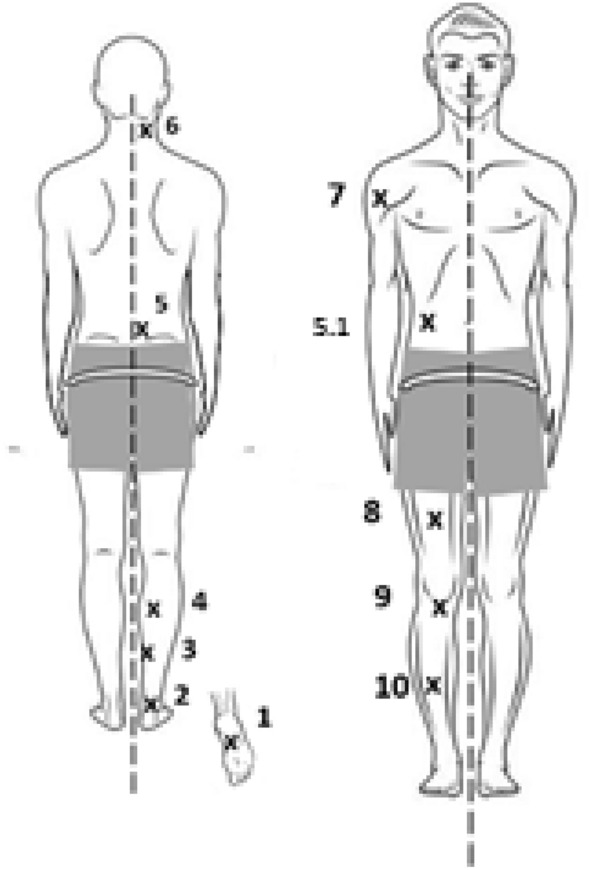
Dorsal (left) and Ventral (right) measurement points (right side only) are shown in body chart.

The MP for this study were based on those for the Myotones project currently ongoing on the ISS^24^, where both technologies are tested at 11 sites (seven of which are assessed in this study). All MPs (all on right side) can be seen below (Fig. 1).

As some of the MPs listed below included in the Myotones study already have well established reliability, only seven measurement points were examined in the present study: MP1, MP2, MP3, MP6, MP7, MP9, and MP10.

#### Participant in prone lying: with roller under the ankle

1. Plantar fascia (MP1)—palpate the fascia (tight band) from the middle arch of the foot up towards the heel until the feel of the band is lost. MP1 is 1 cm down from the base of the heel (confirmed with ultrasound)—ultrasound image taken longitudinally.
2. Achilles tendon (MP2)—thinnest part of the tendon (palpatable using thumb and index finger as “forceps” or “tweezer grip”)—ultrasound image taken longitudinally.
3. Soleus (MP3)—Measure the distance between MP2 and middle of the popliteal crease at the back of the knee. MP3 is located 33% up the back of the calf from MP2, then 3 cm medially—ultrasound image taken longitudinally.
4. Gastrocnemius (MP4)—from MP2, measure 66% up the back of the calf, then 3 cm medially—ultrasound image taken longitudinally.
5. Multifidus lumbar L4 (MP5)—draw the line (tape measure) between the top of hips (Iliac crests of pelvis) to find level on the backbone. From the mid-point on the spine, measure x = 1 cm to the right—ultrasound image taken transversely.
6. Transversus abdominis (TrA; 5.1)—find the Umbilicus (belly button), move laterally until the three lateral abdominal muscles (external oblique, internal oblique, TrA) are seen as parallel (ultrasound only).- ultrasound image taken transversely.

#### Participant in sitting

7. Splenius capitis (MP6)—tape measure between the base of the external occipital protuberance (bump at back of the skull base) and the Acromion (bump on top of shoulder) and mark the centre of the line. Tape measure between C7 and angle of the jaw (mandible) just below the ear and mark where the lines cross. From this intersection, move 3 cm forward from this cross (confirmed with ultrasound).—ultrasound image taken transversely.

#### Participant in supine lying: with roller under the knee

8. Deltoid anterior (MP7)—place a tape measure from the Acromion down to the crease on front of elbow. Put your finger on the tape at the same height as the crease of the armpit and move your finger up 2 cm. To confirm the point, ask the participant to raise their arm, and the muscle should contract under your finger—ultrasound image taken transversely.
9. Rectus femoris (MP8)—tape measure in a line formed between the superior border of the patella and the iliac spine (bump on front of right side of pelvis). From the patella, measure 33% up of the total distance between the above two points—ultrasound image taken transversely.
10. Infrapatellar tendon (MP9)—Locate the inferior edge of the patella in the middle and the tibial tuberosity (bump on top of the tibia or shin bone). Measure 50% of distance between the two points—ultrasound image taken longitudinally.
11. Anterior tibialis (MP10)—measure the distance from the lower edge of the patella to the middle of the ankle joint between the medial and lateral malleoli. Measure 50% of the distance between these two points. Then move 2 cm to the outside of the leg over the muscle belly (Tibialis Anterior)—ultrasound image taken transversely.

### MyotonPRO device

A smartphone-sized, non-invasive digital palpation device (MyotonPRO) was used to assess biomechanical properties of muscles (Myoton AS, Estonia). The MyotonPRO device applies mechanical impulses to the skin (duration 15 ms, force 0.4 N) under a pre-compression force of (0.18 N) on the tissue layer of interest to minimize signal bias from soft tissue overlying muscle and tendon. The device is held perpendicular to the skin ± 5° (checked by MyotonPRO device). The impulses cause damped oscillations of the underlying tissues, which are recorded as parameters for tone (represented by frequency, Hz), stiffness (N/m), and elasticity (Logarithmic Decrement). There is a small mark on the probe to show how far to push down on the skin and once the pre-compression load is met, an indicator light changes from orange to green and the preset impulses are applied automatically. The MyotonPRO device records the coefficient of variation (CV) between the sets of at least five different mechanical impulses per measurement and displays this as a percentage next to each parameter. In the present study a threshold of 3% CV was set, if any parameters were over this threshold the measurement was repeated.

### Ultrasound imaging

Images were taken using a real-time B-mode ultrasound scanner (ORCHEO lite, SONOSCANNER, Paris, France; designed by CNES, CSA and ESA, referred to as the ECHO device) with a 3.5–16.7 MHz linear transducer. The transducer was placed transversely or longitudinally on the skin, depending upon the MP being imaged (see specific sites above), with minimal pressure to avoid compression of the underlying tissues. Ultrasound images were taken in accordance with the directions stated above, with one image taken at each point.

All images were measured later off-line by one investigator (PM), using a Matlab algorithm (written by MW). Ultrasound imaging of musculoskeletal soft tissues is known to be reliable^30,31^ and valid^32,33^ against the gold standard of magnetic resonance imaging (MRI). As with the Myoton technique, standardization of factors influencing recording of images is important^14^.

### Operator experience

The number of years of experience for the three researchers collecting data was: Researcher 1: Ultrasound Imaging 5 years, Myoton technology 5 years; Researcher 2: Ultrasound 13 years, Myoton technology 9 years; Researcher 3: Ultrasound over 30 years, Myoton technology 9 years.

### Experimental procedure

During the first session one operator initially located the anatomical measurement points (MPs) and marked them on the participant lying on a gurney at full relaxation. The measurements were recorded in a logbook for each participant ahead of the next session. Three independent operators blind to the other recordings then undertook the MyotonPRO and ultrasound measurements. Raters were not required to re-mark the participants within the same sessions, so reliability of the data acquisition was evaluated and not the whole Myoton or ultrasound procedure.

For the MyotonPRO recordings, two sets of five impulses were applied as described above. Coefficient of variation measurements for each variable on the device were inspected and if any were higher than 3% the measurement was retaken. All points were recorded twice and a mean of the 10 pulses was taken and used in the analysis. Ultrasound images were taken in accordance with the directions stated above, with one image taken at each point.

For the between days intrarater reliability, participants were invited back a minimum of a week later for the second session (in the afternoons), where only measured by one operator repeated both ultrasound and MyotonPRO measurements, after relocating the same MPs as in the previous session.

### Data analysis

Interrater reliability was assessed using a two-way mixed, single score intraclass correlation coefficient (ICC) (3,1)^34^.

Intrarater reliability between days was assessed two-way mixed, single score intraclass correlation coefficient (ICC) (3,1)^34^.

Intrarater reliability within sessions was assessed using a two-way mixed, average score intraclass correlation coefficient (ICC) (3,2) to compare between the two sets of five impulses.

All statistical tests were performed using SPSS (v 26, Armonk, NY: IBM Corp), with the alpha value set at 0.05.

The guidelines for interpretation of ICC results were taken from Koo and Li^30^ with below 0.5 = poor, between 0.5–0.75: moderate, between 0.75 and 0.90 = good, and above 0.90 excellent.

### Matlab programme for measuring scans

Ultrasound scans were measured with a custom written graphical user interface (GUI) created in Matlab (Mathworks, USA) using bespoke functions (MW). The GUI allowed the investigator to import the bitmap images obtained from the ultrasound scanner. The GUI calibrated the images by determining a scaling factor between the number of pixels and a 1 cm distance obtained from the scale displayed on the side of the ultrasound image. The GUI then allowed measurements to be made on the ultrasound image by the investigator through identifying and clicking on various landmarks. Specific landmarks and measurements are detailed below for each muscle and tendon. Distances were then calculated as the Euclidean distance between landmarks, then converting to centimetres using the scaling factor and then exported to an Excel file.

Scans were calibrated against the scale on the side of the ultrasound scan, marking a measurement of 1 cm with a cursor.

Passive muscle thickness was measured on cross-section planes of ultrasound scans between the muscle’s borders from the bottom of their superior fascia to the top of the inferior fascia.

Tendon/fascia thickness was measured by creating a 1 cm wide box, then creating a line on the top of the structure and a line at bottom. The distance between the two lines was then automatically measured 100 times and the mean thickness measurement was taken.

**Figure 2:**
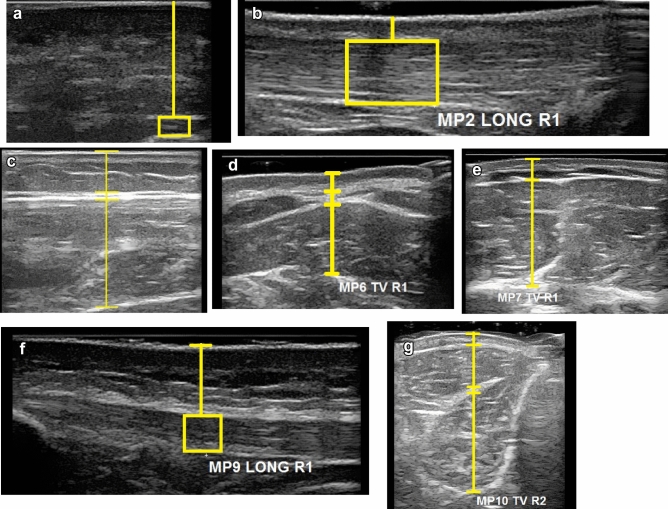
Example of ultrasound, and measurement points for data analysis. (**a**) Plantar Fascia, (**b**) Achilles tendon, (**c**) Soleus, (**d**) Splenius capitis, (**e**) Anterior Deltoid, (**f**) Patellar tendon, (**g**) Anterior tibialis.

Subcutaneous tissue thickness was measured from the top of the skin to the superior border of the muscle fascia (that is regular peripheral body fascia structure with compartment fascia and epimysial layers considered as one structural layer (i.e., muscle fascia) of regional variance however all these structural layers are however below USI image resolution). A second measurement was taken from the superior border of the muscle fascia to its inferior border. The thickness of the fascia was included with fat thickness in the subcutaneous tissue measurement (Fig. 2). The deep foot extensors (MP10 deep foot extensors)- only measured with ultrasound using the same subcutaneous tissue thickness as MP10, but with the muscles measured as one complex all the way to the tibia.

### Minimal detectable change (MDC)

The MDC gives a meaningful and practical assessment of measurement error, providing a single value for each variable in the unit of relevant measure^35^, which is the smallest change in score that occurs due to error and not likely related to chance variation in measurement^36^. The MDC based on the SEM was calculated using the following formula outlined in Haley and Fragala-Pinkham^36^: MDC = 1.96 (z scorelevel of confidence) × √2 × SEM). The formula to calculate SEM was SEM = SD × (√(1 − ICC)) as outlined in Wilken, Rodriguez^35^.

### Bland and Altman

To complement the ICC reliability data, the Bland and Altman test with limits of agreement was used and graphed (Supplementary Table 1)^36^. This test was conducted between each researcher (R1–R2, R2–R3, R1–R3), and for the between day testing.

## Results

The results of the MyotonPRO and Ultrasound are presented below, they range from moderate to excellent, with the vast majority being good or excellent.

### MyotonPRO results

The same day intrarater reliability within session for the MyotonPRO comparing the two sets of five pulses was excellent for all parameters in all sites tested (ranging from 0.96–0.99, Supplementary Table 2) and are not presented in further detail below.

#### Stiffness (N/m)

Interrater reliability within session was excellent or good at all measurement points with the exception of Splenius capitis (MP6 = 0.733) which is moderate (Table 1). Between days intrarater reliability is also excellent or good for all but two measurement points, Splenius capitis (MP6) and Soleus (MP3), which are moderate.

**Table 1:** MyotonPRO stiffness results for intrarater (between days) and interrater reliability, mean stiffness of first session and minimal detectable change (within and between raters).

Measurement point	Interrater reliability	Intrarater reliability	Mean stiffness (N/m) of first session (standard deviation)	Minimal detectable change (N/m) (between days rater 1)	Minimal detectable change (N/m) (interrater)
MP1	0.97	0.96	476 (83)	47.4	40.9
MP2	0.86	0.90	796 (67)	58.8	69.0
MP3	0.95	0.66	368 (88)	140.6	52.0
MP6	0.73	0.57	212 (33)	59.1	47.2
MP7	0.89	0.92	214 (53.3)	40.5	47.7
MP9	0.85	0.98	383 (124.5)	50.9	127.9
MP10	0.93	0.85	573 (81.9)	88.5	61.7

MP1 Plantar Fascia, *MP2* Achilles tendon, *MP3* Soleus, *MP6* Splenius capitis, *MP7* Anterior Deltoid, *MP9* Patellar tendon, *MP10* Anterior tibialis.

#### Tone (frequency)

Most measurement points showed good to excellent interrater reliability with the exception of Achilles tendon (MP2) and Splenius capitis (MP6), both of which have moderate reliability (Table 2). Between day reliability is excellent for plantar fascia (MP1), Soleus (MP3), Tibialis anterior (MP10), good for anterior deltoid (MP7) and Patellar tendon (MP9), and moderate for the Achilles tendon (MP2) and Splenius capitis (MP6). The MDC for each measurement point has also been calculated.

**Table 2:** Myoton frequency results for intrarater (between-days) and interrater reliability, mean frequency of first session and minimal detectable change.

Measurement point	Interrater reliability	Intrarater reliability	Mean frequency (Hz) of first session (standard deviation)	Minimal detectable change (Hz) (between days)	Minimal detectable change (Hz) (interrater)
MP1	0.92	0.85	23.5 (2.6)	3.0	2.0
MP2	0.52	0.52	29.9 (2.4)	4.6	5.0
MP3	0.95	0.90	17.5 (3.6)	3.2	2.1
MP6	0.70	0.51	13.2 (1.0)	2.4	1.6
MP7	0.80	0.75	13.9 (1.8)	2.6	2.3
MP9	0.88	0.77	17.5 (2.6)	3.5	2.5
MP10	0.91	0.80	24.7 (2.8)	3.7	2.5

MP1 Plantar Fascia, *MP2* Achilles tendon, *MP3* Soleus, *MP6* Splenius capitis, *MP7* Anterior Deltoid, *MP9* Patellar tendon, *MP10* Anterior tibialis.

#### Elasticity (decrement)

Interrater reliability was excellent or good at all measurement points with the exception of Solues (MP3; 0.65), which had moderate reliability (Table 3). Between days intrarater reliability was excellent for Splenius capitis (MP6), Anterior Deltoid (MP7), and Anterior tibialis (MP10), and moderate for Plantar Fascia (MP1), Achilles tendon (MP2), Soleus (MP3), and Patellar tendon (MP9).

**Table 3:** Myoton elasticity results for intrarater (between-days) and interrater reliability, mean elasticity of first session and minimal detectable change.

Measurement point	Interrater reliability	Intrarater reliability	Mean elasticity (log decrement) of first session (standard deviation)	Minimal detectable change (log decrement) (between days)	Minimal detectable change (log decrement) (interrater)
MP1	0.90	0.55	1.15 (0.12)	0.23	0.12
MP2	0.77	0.63	0.84 (0.21)	0.33	0.25
MP3	0.65	0.71	1.18 (0.17)	0.27	0.26
MP6	0.92	0.91	1.15 (0.20)	0.17	0.15
MP7	0.77	0.94	0.91 (0.19)	0.13	0.25
MP9	0.85	0.68	0.99 (0.13)	0.21	0.14
MP10	0.87	0.95	0.82 (0.16)	0.09	0.16

MP1 Plantar Fascia, *MP2* Achilles tendon, *MP3* Soleus, *MP6* Splenius capitis, *MP7* Anterior Deltoid, *MP9* Patellar tendon, *MP10* Anterior tibialis.

### Ultrasound imaging results

#### Subcutaneous thickness

Interrater reliability for subcutaneous thickness ranges from excellent Soleus (MP3), Patellar tendon (MP9), and Anterior tibialis (MP10) to moderate Plantar Fascia (MP1), with the majority showing good reliability (Table 4). Intrarater reliability was good or excellent.

**Table 4:** Ultrasound imaging subcutaneous thickness results for interrater and intrarater (between-days) reliability, mean subcutaneous thickness, and minimal detectable change.

Measurement points	Subcutaneous thickness interrater reliability	Subcutaneous thickness Intrarater reliability (between-days)	Mean subcutaneous thickness (mm) of first session (standard deviation)	Subcutaneous thickness MDC between-days (mm)
MP1	0.72	0.88	14.3 (2.2)	2.1
MP2	0.86	0.91	3.4 (1.1)	0.9
MP3	0.96	0.91	11.3 (4.3)	3.8
MP6	0.85	0.90	6.5 (1.8)	1.6
MP7	0.87	0.9	6.3 (4.1)	4.9
MP9	0.90	0.90	6.1 (1.4)	1.3
MP10	0.93	0.86	3.7 (2.0)	2.2

MP1 Plantar Fascia, *MP2* Achilles tendon, *MP3* Soleus, *MP6* Splenius capitis, *MP7* Anterior Deltoid, *MP9* Patellar tendon, *MP10* Anterior tibialis.

#### Muscle/tendon/plantar fascia

Intrarater reliability for muscle/tendon/plantar fascia thickness ranged from excellent Plantar Fascia (MP1), Patellar tendon (MP9), and Anterior tibialis deep muscle group (MP10) to moderate Achilles tendon (MP2). Interrater reliability ranged from excellent Splenius capitis (MP6), Anterior deltoid (MP7), Patellar tendon (MP9), and Anterior tibialis deep muscle group (MP10) to moderate Achilles tendon (MP2), with the majority showing good reliability (Table 5).

**Table 5:** Ultrasound imaging Muscle/tendon/fascia thickness results for interrater and intrarater (between-days) reliability, mean Muscle/tendon/fascia thickness, and minimal detectable change.

Measurement points	Muscle/tendon/fascia thickness interrater reliability	Muscle/tendon/fascia thickness intrarater reliability	Average of first session muscle/tendon/fascia thickness (mm)	Muscle/tendon/fascia MDC between days (mm)
MP1	0.80	0.91	1.85	0.36
MP2	0.74	0.71	4.44	0.83
MP3	0.88	0.82	14.84	3.2
MP6	0.92	0.85	6.9	2.08
MP7	0.90	0.93	19.83	4.17
MP9	0.92	0.90	3.73	0.53
MP10	0.94	0.95	7.06	1.51
MP10 (deep foot extensors)	0.82	0.87	29.38	3.61

MP1 Plantar Fascia, *MP2* Achilles tendon, *MP3* Soleus, *MP6* Splenius capitis, *MP7* Anterior Deltoid, *MP9* Patellar tendon, *MP10* Anterior tibialis.

## Discussion

The purposes of this study have been achieved, which were to: describe the protocol for examining several muscles affected by inactivity using two technologies (MyotonPRO and ultrasound imaging); establish the interrater and intrarater reliability of the measurement techniques; and provide reference values for MDC for use in studies monitoring change over time in people undergoing or recovering from the effects of inactivity. Examples of this include, astronauts during and after microgravity; people in intensive care or inactive with an illness or injury and during rehabilitation; or people living with long-term conditions affecting muscle mass. Reliability for some of the measurement points in the protocol of 11 sites have already been established in the literature in healthy cohorts, so the present study focussed on sites requiring reliability data, including MDC, and for completion in the context of the entire protocol, results from all sites are included in the discussion below. Overall, the majority of measurement points had good or excellent reliability with both the MyotonPRO and ultrasound device, with several found to be moderate, and none poor. It is important to note when interpreting the interrater reliability results that the raters did not re-mark measurement points so only the reliability of the data acquisition was evaluated.

### Reliability of measurements from the MyotonPRO

Same session intrarater reliability was excellent at all points and for all Myoton parameters (ICC 0.96–0.99), which is well established in the literature^8,15,20,37^. This high level of reliability is enabled when, as in the present study, a threshold CV of 3% is used, and any measurements with higher values are discarded and recordings re-taken. Even in Dellalana et al.^11^ where the CV value for the gastrocnemius medialis measurements were 5.5% for stiffness the ICC was 0.96, showing the robustness of the device and the importance of taking a mean of the measurements. It should, however, be noted that a different probe was used in the above study^11^, which focussed on skin stiffness, while the present study focussed on muscle stiffness.

The intrarater reliability between-days was examined by Rater 1 and was excellent to good for all three Myoton parameters at most of the measurement points, with several being moderately reliable. None of the measurement points were fair or poor, which is consistent with the literature on other measurement points^16,30,36,38,39^. The MDCs between sessions are also low (Tables 1, 2, 3).

The lower reliability observed between days compared to measurements during the same session was expected and could be explained by several factors, such as: ability of the operator to find the same testing site on a different day, which was not required for recordings repeated within the same session; subtle changes in the muscle length and tension due to slightly different positioning; different activity prior to testing, or change in temperature etc.^40^. Differences in recordings are unlikely to be due to the device used, which is calibrated by the manufacturer remains consistent when tested on phantom materials. Between-day reliability is necessary to enable monitoring of changes over time to examine the effects of disuse/disease/injury and in response to an intervention. Reliability between days seems to be higher on larger structures that are easier to relocate such as soleus (MP3), gastrocnemius medialis (MP4), multifidus (MP5), and rectus femoris (MP8).

Subcutaneous tissue overlying the tissue of interest (muscle/tendon/fascia) may vary in structure and composition (dermis collagens vs adipose) but also water/fluid content between participants and between different body locations (limbs and trunk). Due to the MyotonPRO device presettings and algorithm, however, short moderate pre-compression of the probe (targeted to soft tissue compression), followed by several stronger compression impulses (targeted to more dense muscle tissue with oscillation signal reflows), does not completely mitigate but minimizes oscillation signal bias from overlying subcutaneous tissue composition^41^.

Interrater reliability was excellent or good for all measurement points, in both the present study and previous studies (Supplementary Table 3). This indicates that the Myoton technique is robust, giving confidence in situations where it is not always possible to have the same operator for each session. Such as in the Schoenrock, Zander^23^ study which was required to used two independent operators due to scheduling. Most studies have involved experienced operators, but reliability has also been examined in novice operators, and has been found to be good^16,37^. Demonstration of reliability in novices was important for using the present protocol in the Myotones project, in which astronauts conduct Myoton recordings (as relative novices) on one another during their 6-month inflight period on the International Space Station^24^.

Not all reliability studies have reported MDCs for all three parameters reported in the present paper, as indicated in Supplementary Table 3.

### Reliability of ultrasound imaging of muscle and related tissues

The present study demonstrated excellent or good intrarater reliability between days (Table 4) for all MPs apart from the thickness of the Achilles tendon, which was still moderate (ICC 0.71). These findings were similar to those of previous studies that also found excellent or good intrarater reliability (Supplementary Table 4). The only other study found to assess intrarater reliability of subcutaneous tissue thickness found excellent results^30^, further supporting the present results.

Interrater reliability was also excellent to good, with the exception of two points (plantar fascia subcutaneous thickness and Achilles thickness, which were still moderate (ICC 0.72 and 0.71 respectively), potentially due to the small measurements involved. This is similar to the results of previous studies assessing ultrasound interrater reliability (Supplementary Table 3)^17,42–45^.

It is common for ultrasound studies to take more than one scan and using the mean value in the analysis (similar to MyotonPRO)^46,47^. Excellent intrarater results within the same session from previous studies^48^ have demonstrated that it is not always necessary to take more than one scan at each measurement point. This fact is very helpful for situations where time restraints make only one measurement feasible, such as astronaut protocols in spaceflight and research projects with high level athletes.

### Relevance of the MPs studied

It is hypothesized that during human body unloading in spaceflight muscle stiffness will decrease particularly in those muscles which are most important for postural support and movement (running, walking) on the ground (e.g. Soleus, Multifidus, vastus lateralis, paraspinal muscles of neck and back), whilst other phasic muscles (such as Gastrocnemius, Rectus abdominis, or shoulder and arm muscles) which are only slightly active if at all during microgravity-induced unloading in spaceflight will be relatively unaffected thus serving as internal control.

### Limitations of the study

A limitation of this study is that the reliability of finding the measurement points of interest has not been established amongst novice users or those who are not familiar with anatomical positions. Between day reliability was also only assessed by one researcher, so other variations could have been present if all the researchers were required to take measurements again. All ultrasound scans were measured by one investigator, so interreliabilty examined the ultrasound acquisition technique of the raters but not the whole technique of obtaining and measuring scans. Furthermore, as same day measurements did not require re-marking of measurement points between raters, the interrater reliability evaluates data acquisition, rather than the full protocol. However, this study design enabled reliability of the actual imaging technique to be examined, which is of value to determine. Whilst effort was made to keep the time of day consistent, this was not always possible, this could have accounted for some of the change in muscle tone, as Basti, Yalçin^29^ states that resting muscle tone can be naturally change across the time of day.

## Conclusions

A protocol for testing muscle biomechanical properties such as tone, stiffness and elasticity (using the MyotonPRO device) and subcutaneous and muscle thickness (using ultrasound imaging) has been described for measuring several sites of the healthy body (female and male) relevant for assessing the effects of inactivity and recovery in relatively young and healthy study participants. Reliability of both technologies was high. Intrarater reliability (same session) for the MyotonPRO was consistently excellent (all ICCs > 0.96) at all measurement points and for each of the three parameters, indicating that only one set of five mechanical impulses (5 × 0.4 N force) are necessary per measurement point.

Overall, interrater reliability of MyotonPRO measurements was excellent to good, with a small number of values moderate. This indicates that any Myoton operators within future protocols in many research laboratories and clinical settings will be able to collect reliable data.

The excellent to moderate intra-rater reliability (between-days) results indicate that the Myoton results for the operator studied (Rater 1) were repeatable.

The ultrasound scans from this study have been shown to be good to excellent except for three points which remained moderate: Intrarater reliability between days thickness of the Achilles tendon (ICC 0.71), Interrater reliability plantar fascia subcutaneous thickness and Achilles tendon thickness, (ICC 0.72 and 0.71 respectively).

Minimal detectable change (MDC) values have been documented for measurement points over particular muscles, tendons and the plantar fascia, for both Myoton and ultrasound measurements, providing a reference source for studies monitoring change over time. Values for MDC available in the literature from measurement points in the Myotones protocol but not included in the present study are also given in the present paper to provide a more complete reference for the protocol.

## Supplementary Information

Supplementary Tables.

## Data Availability

The datasets generated during and/or analysed during the current study are available from the corresponding author on request.
