# Supplementary material for: Protocol and reference values for minimal detectable change of MyotonPRO and ultrasound imaging measurements of muscle and subcutaneous tissue
Source: Sci Rep. 2022 Aug 11;12:13654. doi: 10.1038/s41598-022-17507-2 (PMC9372175; doi:10.1038/s41598-022-17507-2)
Supplement: Supplementary file 1 — Supplementary Tables. [file 41598_2022_17507_MOESM1_ESM.docx]

# Supplementary data

| Supplementary table 1. Bland and Altman results for MP1, 2, 3, 6, 7, 9, and 10 for MyotonPRO (frequency, decrement, and stiffness), and ultrasound (subcutaneous tissue and muscle/tendon/fascia thickness) for R1-R2, R2-R3, R1-R3, and R1-R1 (between days) | | | | | | | | | | | | |
| --- | --- | --- | --- | --- | --- | --- | --- | --- | --- | --- | --- | --- |
| MyotonPRO | | | | | | | | | | | | |
| Frequency | | | | | | | | | | | | |
|  | R1-R2 | | | R2-R3 | | | R1-R3 | | | R1-R1 between days | | |
| Measurement point | Mean | Lower | Upper | Mean | Lower | Upper | Mean | Lower | Upper | Mean | Lower | Upper |
| MP1 | -0.49 | -2.64 | 1.67 | 0.69 | -1.24 | 2.62 | 0.20 | -1.81 | 2.22 | -0.36 | -3.43 | 2.72 |
| MP2 | -0.23 | -5.33 | 4.87 | -0.27 | -5.65 | 5.11 | -0.50 | -5.09 | 4.09 | -0.08 | -4.71 | 4.55 |
| MP3 | -0.19 | -2.24 | 1.86 | 0.14 | -2.11 | 2.40 | -0.19 | -2.24 | 1.86 | -0.14 | -3.39 | 3.12 |
| MP6 | 0.02 | -1.17 | 1.21 | -0.14 | -1.91 | 1.63 | -0.12 | -2.05 | 1.81 | -0.28 | -2.68 | 2.13 |
| MP7 | -0.17 | -2.09 | 1.74 | 0.17 | -2.54 | 2.88 | 0.00 | -2.43 | 2.43 | 0.52 | -2.04 | 3.09 |
| MP9 | -0.05 | -2.49 | 2.39 | -0.05 | -2.63 | 2.53 | -0.10 | -2.76 | 2.57 | 0.37 | -3.21 | 3.95 |
| MP10 | -1.00 | -3.33 | 1.33 | 0.82 | -2.25 | 3.90 | -0.16 | -3.91 | 3.59 | -0.16 | -3.91 | 3.59 |
| Decrement | | | | | | | | | | | | |
|  | R1-R2 | | | R2-R3 | | | R1-R3 | | | R1-R1 between days | | |
| Measurement point | Mean | Lower | Upper | Mean | Lower | Upper | Mean | Lower | Upper | Mean | Lower | Upper |
| MP1 | 0.00 | -0.15 | 0.14 | 0.01 | -0.10 | 0.11 | 0.00 | -0.11 | 0.11 | 0.05 | -0.18 | 0.28 |
| MP2 | 0.02 | -0.19 | 0.22 | 0.03 | -0.21 | 0.26 | 0.04 | -0.27 | 0.36 | -0.02 | -0.36 | 0.32 |
| MP3 | -0.03 | -0.29 | 0.23 | -0.03 | -0.27 | 0.22 | -0.06 | -0.33 | 0.22 | -0.08 | -0.34 | 0.19 |
| MP6 | 0.01 | -0.17 | 0.18 | 0.01 | -0.17 | 0.18 | 0.01 | -0.13 | 0.16 | -0.04 | -0.20 | 0.13 |
| MP7 | -0.03 | -0.12 | 0.06 | 0.03 | -0.28 | 0.33 | 0.00 | -0.30 | 0.29 | 0.00 | -0.30 | 0.29 |
| MP9 | -0.02 | -0.17 | 0.14 | -0.01 | -0.15 | 0.12 | -0.03 | -0.15 | 0.09 | -0.03 | -0.15 | 0.09 |
| MP10 | 0.00 | -0.17 | 0.18 | 0.00 | -0.16 | 0.16 | 0.00 | -0.14 | 0.14 | 0.00 | -0.14 | 0.14 |
| Stiffness | | | | | | | | | | | | |
|  | R1-R2 | | | R2-R3 | | | R1-R3 | | | R1-R1 between days | | |
| Measurement point | Mean | Lower | Upper | Mean | Lower | Upper | Mean | Lower | Upper | Mean | Lower | Upper |
| MP1 | -17.19 | -53.02 | 18.64 | 17.87 | -29.60 | 65.33 | 0.68 | -38.79 | 40.14 | 0.88 | -47.07 | 48.84 |
| MP2 | 2.02 | -79.03 | 83.06 | -1.69 | -47.94 | 44.57 | 0.33 | -77.44 | 78.10 | 5.85 | -53.56 | 65.27 |
| MP3 | -5.56 | -54.28 | 43.15 | 2.60 | -55.13 | 60.32 | -2.97 | -54.48 | 48.54 | -5.18 | -72.68 | 62.32 |
| MP6 | -3.03 | -53.66 | 47.60 | 2.85 | -35.61 | 41.31 | -6.55 | -66.10 | 53.00 | -6.55 | -66.10 | 53.00 |
| MP7 | -1.67 | -44.73 | 41.39 | 1.56 | -53.58 | 56.70 | -0.11 | -46.64 | 46.42 | 8.34 | -32.48 | 49.16 |
| MP9 | 13.11 | -92.09 | 118.31 | -6.83 | -141.89 | 128.24 | 6.28 | -140.07 | 152.64 | 10.00 | -41.60 | 61.60 |
| MP10 | -22.24 | -75.21 | 30.74 | 22.73 | -46.48 | 91.93 | 0.49 | -63.59 | 64.57 | 0.35 | -89.22 | 89.92 |
| Ultrasound | | | | | | | | | | | | |
| Subcutaneous tissue | | | | | | | | | | | | |
|  | R1-R2 | | | R2-R3 | | | R1-R3 | | | R1-R1 between days | | |
| Measurement point | Mean | Lower | Upper | Mean | Lower | Upper | Mean | Lower | Upper | Mean | Lower | Upper |
| MP1 | -0.02 | -0.28 | 0.23 | 0.02 | -0.29 | 0.34 | 0.00 | -0.39 | 0.39 | -0.07 | -0.28 | 0.14 |
| MP2 | -0.01 | -0.12 | 0.11 | 0.00 | -0.09 | 0.10 | 0.00 | -0.10 | 0.10 | 0.00 | -0.09 | 0.09 |
| MP3 | -0.01 | -0.33 | 0.32 | -0.01 | -0.19 | 0.17 | -0.01 | -0.28 | 0.25 | -0.03 | -0.45 | 0.39 |
| MP6 | 0.01 | -0.20 | 0.22 | 0.01 | -0.19 | 0.20 | 0.02 | -0.15 | 0.18 | -0.01 | -0.17 | 0.16 |
| MP7 | -0.03 | -0.32 | 0.26 | 0.01 | -0.47 | 0.49 | -0.02 | -0.52 | 0.49 | -0.10 | -0.54 | 0.33 |
| MP9 | -0.02 | -0.15 | 0.12 | 0.01 | -0.11 | 0.13 | 0.00 | -0.14 | 0.14 | -0.05 | -0.17 | 0.08 |
| MP10 | -0.05 | -0.20 | 0.10 | 0.04 | -0.11 | 0.19 | -0.02 | -0.18 | 0.14 | -0.02 | -0.24 | 0.20 |
| Muscle/tendon/fascia thickness | | | | | | | | | | | | |
|  | R1-R2 | | | R2-R3 | | | R1-R3 | | | R1-R1 between days | | |
| Measurement point | Mean | Lower | Upper | Mean | Lower | Upper | Mean | Lower | Upper | Mean | Lower | Upper |
| MP1 | 0.00 | 0.00 | -0.05 | 0.00 | -0.05 | 0.04 | 0.00 | -0.05 | 0.06 | 0.01 | -0.03 | 0.04 |
| MP2 | 0.02 | -0.09 | 0.13 | -0.01 | -0.07 | 0.05 | 0.01 | -0.08 | 0.09 | 0.01 | -0.07 | 0.10 |
| MP3 | 0.00 | -0.27 | 0.26 | 0.02 | -0.24 | 0.27 | 0.01 | -0.29 | 0.32 | 0.00 | -0.33 | 0.32 |
| MP6 | 0.00 | -0.12 | 0.13 | 0.00 | -0.19 | 0.19 | 0.01 | -0.17 | 0.18 | 0.00 | -0.21 | 0.21 |
| MP7 | -0.03 | -0.67 | 0.60 | 0.09 | -0.55 | 0.74 | 0.06 | -0.40 | 0.51 | 0.00 | -0.42 | 0.42 |
| MP9 | 0.00 | -0.05 | 0.05 | 0.00 | -0.04 | 0.04 | 0.00 | -0.04 | 0.05 | 0.00 | -0.05 | 0.06 |
| MP10 | 0.00 | -0.33 | 0.34 | 0.02 | -0.44 | 0.49 | 0.03 | -0.47 | 0.53 | 0.03 | -0.33 | 0.40 |

| Supplementary table 2 . Intrarater reliability for within session for the MyotonPro, stiffness, frequency, elasticity | | | |  |
| --- | --- | --- | --- | --- |
|  |  |  |  |  |
| Measurement point | stiffness | frequency | elasticity |  |
| MP1 | 0.99 | 0.99 | 0.98 |  |
| MP2 | 0.96 | 0.96 | 0.99 |  |
| MP3 | 1.00 | 1.00 | 0.97 |  |
| MP6 | 0.98 | 0.97 | 0.97 |  |
| MP7 | 0.99 | 0.99 | 0.99 |  |
| MP9 | 0.98 | 0.99 | 0.98 |  |
| MP10 | 1.00 | 0.99 | 0.99 |  |

MP1=Planta Fascia, MP2=Achilles tendon, MP3=Soleus, MP6=Splenius capitis, MP7=Anterior Deltoid, MP9=Patellar tendon, MP10= Anterior tibialis

| study | measurement point | type of reliability | Stiffness ICC | Mean Stiffness (MDC) | Frequency ICC | Mean frequency (MDC) | Decrement ICC | Mean decrement (MDC) |
| --- | --- | --- | --- | --- | --- | --- | --- | --- |
| Dellalana [1] | Gastrocnemius medialis | interrater same day | 0.78 | 378.3 (99.6) |  |  |  |  |
|  |  | intrarater reliability same day | 0.96 | 372.7 (50.3) |  |  |  |  |
| Feng [2] | Gastrocnemius medialis | Intrarater reliability between days | 0.787 | 314.32 (28.19) |  |  |  |  |
| Kelly [3] | Gastrocnemius medialis | intrarater reliability same day | 1 | 326.2 (SD or SEM not reported) |  |  |  |  |
| Taş [4] | Gastrocnemius medialis | interrater same day | 0.98 | 374 (41) |  |  |  |  |
|  |  | intrarater reliability between days | 0.91 | 367 (79) |  |  |  |  |
| Chuang [5] | Gastrocnemius medialis | interrater reliability same day | 0.88 | 325.9 (58.15**†**) | 0.91 | 16.2 (2.38**†**) | 0.86 | 1.6 (0.44**†**) |
| Lohr [6] | Erector spine | interrater reliability same day | 0.97 | 302.40 (45.5) | 0.99 | 15.65 (0.94) | 0.95 | 1.25 (0.47) |
|  |  | interrater reliability between days | 0.96 | 303.62 (44.4) | 0.92 | 15.61 (2.11) | 0.86 | 1.26 (0.25) |
| Kelly [3] | Erector spine | Intrarater reliability same day | 0.98 | 289.4 (SD or SEM not reported) |  |  |  |  |
| Hu [7] | Multifidus | Intrarater reliability between days | 0.96 | 261.4 (22.8) | 0.96 | 14.8 (0.6) |  |  |
| Mullix [8] | Rectus femoris | Intrarater reliability same day | 0.99 | 275.5 (28.8) | 0.99 | 15.45 (1.34) | 0.99 | 1.33 (0.19) |
|  |  | Intrarater reliability between days | 0.83 | 275.5 (23.8) | 0.81 | 15.45 (1.29) | 0.87 | 1.30.5 (0.21) |
| Aird [9] | Rectus femoris | Intrarater reliability same day | 0.97 | (13.7) | 0.99 | (0.4) | 0.99 | (0.1) |
|  |  | Intrarater reliability between days | 0.82 | 314.85 (29.7) | 0.77 | 15.9 (1.9) | 0.79 | 1.7 (0.4) |
| Agyapong-Badu [10] | Rectus femoris | Intrarater reliability same day | 0.99 | 262.6 (13.39) | 0.99 | 15.0 (0.58) | 0.97 | 1.2 (0.08) |
|  |  | Intrarater reliability between days (young participants) | 0.93 | 270 (34) | 0.94 | 15.15 (1.25) | 0.68 | 1.3 (0.31) |
|  |  | Intrarater reliability between days (old participants) | 0.9 | 310 (34.8) | 0.92 | 15.45 (1.49) | 0.76 | 1.65 (0.34) |

Supplementary table 3. MyotonPRO reliability results from the literature, ICC values, mean and MDC (MP4, 5 and 8)

**† MDC calculated from given SD or SEM. Missing values were not presented in studies**

| Supplementary table 4. Ultrasound imaging reliability results from literature, ICC values, mean and MDC, and number of images taken | | |  |  |  |
| --- | --- | --- | --- | --- | --- |
| Study | Measurement point | Type of reliability | ICC | Mean mm (MDC) | Number of images taken |
| Raj [11] | Gastroc | Interrater | 0.97 | 17.9-18.0 (1.42*) | 3 |
| Cho [12] | Gastroc | Interrater | 0.967-0.973 | 13.9-14.0 (0.38*) | 1 |
|  |  | Intrarater between days | 0.982-0.992 | 13.9-14.0 (0.25*) | 1 |
| Wilson [13] | TrA | Interrater | 0.99 | 3.8 (0.4) | 1 |
|  |  | Intrarater between days | 0.99 | 3.8 (0.5) | 1 |
| Stetts [14] | TrA | Interrater | 0.92 | 5.4 (0.28*) | 3 |
|  |  | Intrarater between days | 0.97 | 5.6 (0.83*) | 1 |
| Norasteh [15] | TrA | Interrater | 0.81 | 4.36 (1.244) | 1 |
| Rankin [16] | TrA | Intrarater between days | 0.96-0.99 | 4.5 (SD or SEM not reported) | 1 |
| Wilson [13] | Multifidus (L5/S1) | interrater | 0.86 | 28.9 (5.5) | 1 |
|  | Multifidus (L4/5) | Intrarater between days | 0.95 | 32.6 (3.5) | 1 |
| Sions [17] | Multifidus (L4/5) | interrater | 0.88-0.90 | 29.3-33.8 (4.0-7.3) | 3 |
|  |  | Intrarater between days | 0.85-0.94 | 28.7-33.4 (3.4-6.2) | 3 |
| Koppenhaver [18] | Multifidus (L4/5) | Intrarater between days | 0.88 (0.93) | (SD or SEM not given) | 1 (6) |
| Wong [19] | Multifidus (L4/5) | intrarater same day | 0.99 | 26.1 (1.081) | 3 |
| Hadda [20] | Rectus femoris | Interrater | 0.992 | (SD or SEM not given) | 3 |
|  |  | Intrarater between days | 0.835-0.925 | (SD or SEM not given) | 1 |
| Agyapong-Badu [21] | Anterior thigh | Intrarater between days | 0.88-0.89 | 20-38.7 (5.5-5.9mm) | 1 |
|  | Anterior thigh subcutaneous thickness | Intrarater between days | 0.97 | 7.8-15.8 (2.28-2.49) | 1 |
| Birtles [22] | anterior thigh | intrarater same day | 0.95-0.99 | (SD or SEM not given) | 1 |
|  |  | Intrarater between days | 0.96 | (SD or SEM not given) | 1 |
| Bemben [23] | anterior thigh | interrater between days | 0.72-0.88 | (SD or SEM not given) | Unknown |
| Thoirs [24] | anterior thigh | interrater same day | 0.9 | 36.1 (6.44mm*) | 1 |
| Filippo [25] | Anterior thigh | Interrater | 0.98 | , (2.74) | 1 |
|  | Anterior thigh non-contractile tissue | Interrater | 0.78 | , (4.29) | 1 |
|  | Anterior thigh | Intrarater between days | 0.96 | , (3.6) | 1 |
|  | Anterior thigh non-contractile tissue | Intrarater between days | 0.98 | , (1.83) | 1 |
| Thomaes [26] | Rectus femoris | intrarater between days | 0.97 | 16.01 (2.4) | 5 |

1. Dellalana, L.E., et al., *Reproducibility of the durometer and myoton devices for skin stiffness measurement in healthy subjects.* Skin Research and Technology, 2018.

2. Feng, Y.N., et al., *Assessing the elastic properties of skeletal muscle and tendon using shearwave ultrasound elastography and MyotonPRO.* Scientific Reports, 2018. **8**(1): p. 17064.

3. Kelly, J.P., et al., *Characterization of tissue stiffness of the infraspinatus, erector spinae, and gastrocnemius muscle using ultrasound shear wave elastography and superficial mechanical deformation.* Journal of Electromyography and Kinesiology, 2018. **38**: p. 73-80.

4. Taş, S. and Y. Salkın, *An investigation of the sex-related differences in the stiffness of the Achilles tendon and gastrocnemius muscle: Inter-observer reliability and inter-day repeatability and the effect of ankle joint motion.* The Foot, 2019. **41**: p. 44-50.

5. Chuang, L.L., C.Y. Wu, and K.C. Lin, *Reliability, Validity, and Responsiveness of Myotonoinetric Measurement of Muscle Tone, Elasticity, and Stiffness in Patients With Stroke.* Archives of Physical Medicine and Rehabilitation, 2012. **93**(3): p. 532-540.

6. Lohr, C., et al., *Reliability of tensiomyography and myotonometry in detecting mechanical and contractile characteristics of the lumbar erector spinae in healthy volunteers.* European journal of applied physiology, 2018: p. 1-11.

7. Hu, X., et al., *Quantifying paraspinal muscle tone and stiffness in young adults with chronic low back pain: a reliability study.* Scientific reports, 2018. **8**(1): p. 1-10.

8. Mullix, J., M. Warner, and M. Stokes, *Testing muscle tone and mechanical properties of rectus femoris and biceps femoris using a novel hand held MyotonPRO device: relative ratios and reliability.* Working Papers in Health Sciences, 2012. **1**(1): p. 1-8.

9. Aird, L., D. Samuel, and M. Stokes, *Quadriceps muscle tone, elasticity and stiffness in older males: reliability and symmetry using the MyotonPRO.* Archives of gerontology and geriatrics, 2012. **55**(2): p. e31-e39.

10. Agyapong-Badu, S., et al., *Measurement of ageing effects on muscle tone and mechanical properties of rectus femoris and biceps brachii in healthy males and females using a novel hand-held myometric device.* Archives of gerontology and geriatrics, 2016. **62**: p. 59-67.

11. Raj, I.S., S.R. Bird, and A.J. Shield, *Reliability of ultrasonographic measurement of the architecture of the vastus lateralis and gastrocnemius medialis muscles in older adults.* Clinical physiology and functional imaging, 2012. **32**(1): p. 65-70.

12. Cho, K.H., H.J. Lee, and W.H. Lee, *Reliability of rehabilitative ultrasound imaging for the medial gastrocnemius muscle in poststroke patients.* Clinical physiology and functional imaging, 2014. **34**(1): p. 26-31.

13. Wilson, A., et al., *Measuring ultrasound images of abdominal and lumbar multifidus muscles in older adults: A reliability study.* Manual Therapy, 2016. **23**: p. 114-119.

14. Stetts, D.M., et al., *A rehabilitative ultrasound imaging investigation of lateral abdominal muscle thickness in healthy aging adults.* Journal of Geriatric Physical Therapy, 2009. **32**(2): p. 16-22.

15. Norasteh, A., et al., *Reliability of B-mode ultrasonography for abdominal muscles in asymptomatic and patients with acute low back pain.* Journal of Bodywork and Movement Therapies, 2007. **11**(1): p. 17-20.

16. Rankin, G., M. Stokes, and D.J. Newham, *Abdominal muscle size and symmetry in normal subjects.* Muscle & nerve, 2006. **34**(3): p. 320-326.

17. Sions, J.M., et al., *Ultrasound imaging: intraexaminer and interexaminer reliability for multifidus muscle thickness assessment in adults aged 60 to 85 years versus younger adults.* journal of orthopaedic & sports physical therapy, 2014. **44**(6): p. 425-434.

18. Koppenhaver, S.L., et al., *The effect of averaging multiple trials on measurement error during ultrasound imaging of transversus abdominis and lumbar multifidus muscles in individuals with low back pain.* journal of orthopaedic & sports physical therapy, 2009. **39**(8): p. 604-611.

19. Wong, A.Y., E. Parent, and G. Kawchuk, *Reliability of 2 ultrasonic imaging analysis methods in quantifying lumbar multifidus thickness.* journal of orthopaedic & sports physical therapy, 2013. **43**(4): p. 251-262.

20. Hadda, V., et al., *Intra-and inter-observer reliability of quadriceps muscle thickness measured with bedside ultrasonography by critical care physicians.* Indian journal of critical care medicine: peer-reviewed, official publication of Indian Society of Critical Care Medicine, 2017. **21**(7): p. 448.

21. Agyapong-Badu, S., et al., *Anterior thigh composition measured using ultrasound imaging to quantify relative thickness of muscle and non-contractile tissue: a potential biomarker for musculoskeletal health.* Physiological measurement, 2014. **35**(10): p. 2165.

22. Birtles, D., et al., *Effect of eccentric exercise on patients with chronic exertional compartment syndrome.* European journal of applied physiology, 2003. **88**(6): p. 565-571.

23. Bemben, M.G., *Use of diagnostic ultrasound for assessing muscle size.* Journal of Strength and Conditioning Research, 2002. **16**(1): p. 103-108.

24. Thoirs, K. and C. English, *Ultrasound measures of muscle thickness: intra‐examiner reliability and influence of body position.* Clinical physiology and functional imaging, 2009. **29**(6): p. 440-446.

25. Filippo, M., et al., *Inter-rater and intra-rater reliability of ultrasound imaging for measuring quadriceps muscle and non-contractile tissue thickness of the anterior thigh.* Biomedical Physics & Engineering Express, 2019. **5**(3): p. 037002.

26. Thomaes, T., et al., *Reliability and validity of the ultrasound technique to measure the rectus femoris muscle diameter in older CAD-patients.* BMC Medical Imaging, 2012. **12**(1): p. 7.
